# Methanol extract of Melastoma malabathricum leaves exerted antioxidant and liver protective activity in rats

**DOI:** 10.1186/1472-6882-13-326

**Published:** 2013-11-23

**Authors:** Siti Syariah Mamat, Mohamad Fauzi Fahmi Kamarolzaman, Farhana Yahya, Nur Diyana Mahmood, Muhammad Syahmi Shahril, Krystal Feredoline Jakius, Norhafizah Mohtarrudin, Siew Mooi Ching, Deny Susanti, Muhammad Taher, Zainul Amiruddin Zakaria

**Affiliations:** 1 Department of Biomedical Science, Faculty of Medicine and Health Sciences, Universiti Putra Malaysia, 43400 UPM Serdang, Selangor, Malaysia; 2 Department of Pathology, Faculty of Medicine& Health Sciences, Universiti Putra Malaysia, 43400 UPM, Serdang, Selangor, Malaysia; 3 Department of Family Medicine, Faculty of Medicine and Health Sciences, 43400 UPM Serdang, Selangor, Malaysia; 4 Department of Biomedical Science, Kulliyyah of Science, International Islamic University Malaysia, Jl Sultan Ahmad Shah, Bandar Indera Mahkota, 25200 Kuantan, Pahang, Malaysia; 5 Department of Pharmaceutical Technology, Kulliyyah of Pharmacy, International Islamic University Malaysia, Jalan Istana, Bandar Indera Mahkota, 25200 Kuantan, Pahang, Malaysia

**Keywords:** *Melastoma malabathricum*, Melastomaceae, Methanol extract, Hepatoprotective activity, Antioxidant activity, Flavonoids

## Abstract

**Background:**

*Melastoma malabathricum* L. (Melastomaceae) is a small shrub with various medicinal uses. The present study was carried out to determine the hepatoprotective activity of methanol extract of *M. malabathricum* leaves (MEMM) against the paracetamol-induced liver toxicity in rats model.

**Methods:**

The respective chemicals and herbal solutions (10% DMSO, 200 mg/kg silymarin or MEMM (50, 250 and 500 mg/kg)) were administered orally to rats once everyday for 7 days followed by the hepatotoxicity assay. The blood samples and livers were collected and subjected to biochemical and microscopical analysis. Prior to the hepatoprotective study, MEMM was subjected to determination of the total phenolic content (TPC) and the antioxidant properties using several standard assays (e.g. 2, 2-diphenyl-1-picrylhydrazyl- and superoxide anion- radical scavenging assay, and oxygen radical absorbance capacity assay).

**Results:**

MEMM exerted significant (p < 0.05) and high antioxidant activity in which high TPC was recorded; while in the hepatotoxicity study, the extract exhibited significant hepatoprotective effects against the paracetamol-induced hepatotoxic model. The results observed for serum liver enzymes (ALT, ALP and AST) as well as the microscopic observations and microscopic scoring supported the hepatoprotective potential of MEMM. The phytochemical and HPLC analysis of MEMM demonstrated the presence of flavonoids as its major constituents.

**Conclusions:**

The MEMM-induced hepatoprotective activity could be allied partly to its antioxidant activity and the presence of flavonoids.

## Background

Liver is the largest organ in the human body and is a key organ of metabolism [1,2]. Despite its considerable regenerative capacity, continuous and various exposures to xenobiotics, environmental pollutants, and chemotherapeutic agents could suppress and possibly overcome the natural protective mechanisms of the liver, leading to liver malfunction and later if it is not treated properly leads to injury. Despite the remarkable progresses in conventional medical therapies in the last 20 years, drugs available for the treatment of liver diseases were often limited in efficacy and could have triggered various unwanted side effects when compared to other medical therapies for liver diseases which were often difficult to handle [3]. Moreover, some of these modern hepatoprotective drugs did not offer protection against injury to the vital organ despite their direct or indirect action to improve the liver function. In response to these factors that limit the use of conventional drugs, attempts were continuously made by scientists all over the world working in the area of hepatoprotective drug discovery to identify new sources of agents with potential liver protective activity [4,5].

Interestingly, in line with increase in patients attempt to use complementary and alternative medicines, particularly herbal/plant-based therapies, to cure various diseases, efforts are increasingly being carried out by scientists to investigate the hepatoprotective potential of various medicinal plants. One of the medicinal plants that have been widely used in the Malay traditional medicine is *Melastoma malabathricum* L., which is locally known as “*Senduduk*”*.* This small shrub belongs to the family *Melastomaceae* and is native to tropical and temperate Asia, including Malaysia, and the Pacific Islands. Despite being a well-known herb in Malaysia wherein its leaves, shoots and roots are prepared in various ways to treat various types of diseases (i.e. to treat cuts or wounds, puerperal infections, high blood pressure, diabetes, dysentery, diarrhea, piles, leucorrhea, epilepsy, ulcers, gastric ulcers, scar, skin diseases, pimple and black spot at skin, hemorrhoidal bleeding, rheumatism, arthritis, prolonged fever, cancer and tenderness in the legs, to stop bleeding, to prevent scarring from smallpox, and to relieve a toothache), attempts to scientifically investigate and confirm those claims are however lacking [6]. Scientifically, various types of extracts from different parts of *M. malabathricum* have been prepared and tested using a variety of *in vitro* and *in vivo* test models. The plant has been reported to possess various types of pharmacological activities (i.e. antibacterial, antiviral, antiparasitic, cytotoxicity, anticoagulant, platelet-activating factor inhibitory, wound healing, antiulcer, antidiarrheal, antivenom, anti-inflammatory, antinociceptive and antipyretic) at different doses or concentrations [7].

Various phytochemical constituents have been isolated and identified from various parts and extracts of *M. malabathricum*[8]. Focusing, particularly, on the leaves, several bioactive compounds have been identified from different types of extracts, namely: i) 70% acetone extract – isoquercitrin 6*″*-O-gallate, malabathrin-A, -B, -C, -D, -E and -F, 1,4,6-tri*-O-*galloyl-*β*-D-glucoside, 1,2,4,6-tetra*-O-*galloyl-*β*-D-glucoside, strictinin, casuarictin, pedunculagin, nobotanin-B, -D, -G, -H and -J, pterocarinin C, new complex tannins in which an ellagitannin and a flavan-3-ol are bound by a C-glycosidic linkage belonging to type II + tannins, casuarinin, (−)-epicatechin gallate, (−)-epicatechin, stachyurin, procyanidin-B2 and -B5, stenophyllanins A and B, alienanin B, and brevifolincarboxylic acid; ii) methanol extract – ursolic acid, 2-hydroxyursolic acid and asiatic acid, as well as glycerol-1,2-dilinolenyl-3*-O-β*-Dgalactopyranoside and glycerol 1,2-dilinolenyl-3*-O-* (4,6-di*-O-*isopropylidene)-*β*-D-galactopyranoside, 2,5,6-trihydroxynaphtoic carbonic acid, methyl-2,5,6-trihydroxynaphtalene carbonate, flavonol glycoside derivative, quercitrin and kaempferol-3*-O-*(2*′*,6*′*-di*-O-p-trans*-coumaroyl)-*β*-glucoside; iii) hexane fraction of methanol extract – *β*-sitosterol, *α*-amyrin, uvaol, quercetin, quercitrin, rutin, and sitosterol-3*-O-β*-D-glucopyranoside; iv) 90% aqueous methanolic extract – ursolic acid, 2*α*-hydroxyursolic acid and asiatic acid, *β*-sitosterol 3*- O-β*-Dglucopyranoside, glycerol 1,2-dilinolenyl-3*-O-β*-D-galactopyranoside and glycerol 1,2-dilinolenyl-3*-O-*(4,6*-O-*isopropylidene)-*β*-D-galactopyranoside; v) ethyl acetate extract – 2,5,6-trihydroxynaphtoic carbonic acid, methyl-2,5,6-trihydroxynaphtalene carbonate, and flavonol glycoside derivative, quercetin and quercitrin; and vi) hexane extract – 2,5,6-trihydroxynaphtoic carbonic acid, methyl-2,5,6-trihydroxynaphtalene carbonate, and flavonol glycoside derivative, *α*-amyrin, patriscabatrine and auranamide.

Based on the previously demonstrated anti-inflammatory activity of *M. malabathricum* and the reports relating, at least, the anti-inflammatory and antioxidant activities to the hepatoprotective mechanism [9-11], the present study was carried out with an attempt to explore the antioxidant and hepatoprotective activities of *M. malabathricum*. It is postulated that *M. malabathricum* leaves extract, which possesses the anti-inflammatory activities, will also exert antioxidant and hepatoprotective activities. Therefore, the aim of the present study was to determine the hepatoprotective activity of methanol extract of *M. malabathricum* (MEMM) using the paracetamol-induced liver damage in rats as the animal model. In addition, the antioxidant activity, phytochemical content and HPLC profile of MEMM were also verified to support the hepatoprotective potential of the extract.

## Methods

### Collection of plant material

The leaves of *M. malabathricum* were continuously collected from their natural habitat in Serdang, Selangor, Malaysia between September 2011-Jun 2012 and identified by comparison with specimens available at the Herbarium of the Laboratory of Natural Products, IBS, UPM, Serdang, Selangor, Malaysia. Sample of the plant was also deposited at the same herbarium (voucher specimen, ACP 0017).

### Preparation of plant extract

The plant extraction procedure was carried out according to the previously described method [12]. Briefly, the leaves were dried under shade at room temperature for at least 7 days, segregated, and pulverized by mechanical grinder to form coarse powder. The coarse powder of air-dried leaves was subjected to methanol extraction whereby 40 g of powder leaves were macerated in 800 ml of methanol for 72 hours in the ratio of 1:20 (w/v). The methanol supernatant obtained was filtered sequentially using cloth filter, cotton wool and Whatman No. 1 filter paper, collected and then evaporated until dryness under reduced pressure (204 mbar) at the temperature of 40°C (Buchi Rotavapor® R210/215, Switzerland). On the other hand, the residue was also collected and subjected to the similar extraction and evaporation processes for another two times [7]. At the end of the evaporation process, the amount of crude dried MEMM obtained was 7.45 g and the percentage of yield was approximately 18.6%.

### Chemicals

Paracetamol, silymarin, nitroblue tetrazolium salt (NBT), phenazine methosulfate (PMS), 2,2′-azobis (2-amidinopropane) dihydrochloride (AAPH), Trolox (6-hydroxy-2, 5, 7, 8-tetramethylchroman-2-carboxylic acid), a water-soluble analog of vitamin E, and β-phycoerithrin, were purchased from Sigma-Aldrich Co. (St. Louis, MO, USA) and used in the present study. All other chemicals and reagents used were of analytical grade.

### Animals

Male Sprague Dawley rats (180–200 g; 8–10 weeks old) and male ICR mice (25–30 g; 5–7 weeks old) were obtained from the Veterinary Animal Unit, Faculty of Veterinary Medicine, Universiti Putra Malaysia (UPM), Malaysia and kept under room temperature (27 ± 2°C; 70 – 80% humidity; 12 h light/dark cycle) in the Animal Holding Unit (UPM). They were supplied with food and water *ad libitum* from the beginning of the experiments. The study protocol of the present study was approved by the Animal House and Use Committee, Faculty of Medicine and Health Sciences, UPM (Ethical approval no.: UPM/FPSK/PADS/BR-UUH/00449). The rats were handled in accordance with current UPM guidelines for the care of laboratory animals and the ethical guidelines for investigations of experimental pain in conscious animals. All experiments were conducted between 09.30 and 18.30 h to minimize the effects of environmental changes.

### Pharmacological studies of MEMM

#### Antioxidant activity of MEMM

In an attempt to determine the antioxidant potential of MEMM, the total phenolic content (TPC), 2, 2-diphenyl-1-picrylhydrazyl (DPPH) radical scavenging activity, superoxide anion radical scavenging and oxygen radical absorbance capacity (ORAC) of MEMM was determined according to the respective method of [13-16] but with slight modifications. Detailed procedures for TPC and DPPH assays were as reported by Zakaria et al. [12].

#### Superoxide anion radical scavenging

Measurement of superoxide anion radicals scavenging activity of MEMM was based on the method described by Liu et al. [15]. Superoxide radicals are generated in PMS – NADH systems by oxidation of NADH and assayed by the reduction of NBT. In these experiments, the superoxide radicals were generated in 3 ml of Tris–HCl buffer (16 mM, pH 8.0) containing 1 ml of NBT (50 μM), 1 ml NADH (78 μM) and MEMM (200 μg/ml). The reaction was started by adding 1 ml of PMS solution (10 μM) to the mixture. The reaction mixture was incubated at 25°C for 5 min; the absorbance was read at 560 nm using a spectrophotometer (Schimadzu UV–vis 1700) against blank samples using L-ascorbic acid as a control. The decreased absorbance of the reaction mixture indicated increasing superoxide anion scavenging activity. The percentage inhibition of superoxide anion generation was calculated using the following formula:

$$\%\text{inhibition}=\left[\left(A_{0}‒A_{1}\right)/A_{0}\right]\times 100$$

where A_0_ was the absorbance of the control (L-ascorbic acid), and A_1_ was the absorbance in the presence of MEMM or standards.

#### Oxygen radical absorbance capacity (ORAC)

The microplate fluorescence reader with an excitation wavelength of 540 nm and an emission wavelength of 565 nm was used [16]. The 75 μL AAPH (160 mM), 150 μL β-PE (68 mg/L), and 20 μM 6-hydroxy-2,5,7,8-tetra-methylchroman-2-carboxylic acid (Trolox) were prepared in 75 mM phosphate buffer (pH 7.0). AAPH and Trolox were prepared fresh while β-PE was prepared earlier and kept at 4°C in dark condition. Trolox standard was diluted in the PBS to give 20 μM working solutions. To the 96-well plates (Nunc, Thermo Scientific) 150 μL of β-PE was added followed by 25 μL of trolox, buffer (blank), or 150 μL MEMM (200 μg/ml), and lastly, 25 μL AAPH was injected into the microplate reader via injector. ORAC values were calculated based on net area under the curve (AUC) obtained by subtracting the AUC of the blank from that of MEMM and compared to the Trolox standards curve. The antioxidant capacity (ORAC) related to trolox is calculated as:

$$\begin{array}{ll} \text{ORAC}\;\text{value} & =\left[\left(\mathrm{AU}C_{\text{sample}}‒\mathrm{AU}C_{\text{blank}}\right)/\left(\mathrm{AU}C_{\text{Trolox}}‒\mathrm{AU}C_{\text{blank}}\right)\right]\\ & \;\times \left[\left(\text{molarity}\;\mathrm{of}\;\text{Trolox}\right)/\left(\text{molarity}\;\mathrm{of}\;\text{sample}\right)\right]. \end{array}$$

#### Hepatoprotective activity of MEMM

The hepatoprotective activity of MEMM was determined using paracetamol-induced hepatotoxicity model, of which rats were used [17]. The animals were fasted for 48 hours prior to the experiment but allowed access to distilled water (dH_2_O) *ad libitum* under standard laboratory conditions. After 48 hours, each group of rats (Group II-VI; n = 6) received the respective dose of chemical (10% DMSO) or herbal (200 mg/kg silymarin or 50, 250 or 500 mg/kg MEMM) solutions orally once daily for 7 consecutive days. The doses of MEMM were selected based on the single dose (5000 mg/kg) acute toxicity study performed in the previous studies. The MEMM did not cause any signs of toxicity at the dose of 5000 mg/kg according to the results shown by all the blood parameters and serum biochemical analysis. However, this dose was found to cause significant reduction of the rats’ body weight. Therefore, the doses range of MEMM (50, 250, 500 mg/kg) used in the present study was selected based on the 100, 20 and 10 folds reduction of the dose 5000 mg/kg used in the toxicity study. The oral administration of paracetamol was performed 3 hours after the administration of the last chemical or herbal solutions on the 7^th^ day. As for Group I, the rats were pretreated with 10% DMSO for 7 consecutive days followed by the administration of 10% DMSO on the 7^th^ day. The animals were divided into 6 groups and treated with chemical or herbal (DMSO, silymarin or MEMM) solutions as described below.

• Group I received 10% DMSO + 10% DMSO.

• Group II received 10% DMSO + paracetamol.

• Group III received 200 mg/kg silymarin + paracetamol

• Pre-treatment groups;

• Group IV received 50 mg/kg MEMM + paracetamol,

• Group V received 250 mg/kg MEMM + paracetamol and;

• Group VI received 500 mg/kg MEMM + paracetamol.

Forty eight (48) hours after the procedure induced hepatic injury, the animals were anesthetized using diethyl ether and the blood was withdrawn for biochemical analysis. The animals were then sacrificed by cervical dislocation and the liver was removed for histopathological studies according to previously published method [17].

### Biochemical analysis of the liver serum enzymes level

Biochemical parameters were assayed according to the standard methods as described by Yahya et al. [17]. The alanine aminotransferase (ALT), alkaline phosphate (ALP), aspartate aminotransferase (AST) serum level were measured using the Hitachi 902 Automatic Chemical Analyser.

### Histopathology investigation of the liver tissue pretreated and untreated with MEMM

The liver tissue was dissected and processed according to the method of El-Beshbishy et al. [18] but with slight modifications. The liver tissue which was dissected out was subsequently fixed in the 10% formalin, dehydrated in gradual ethanol (50-100%), cleared in xylene and embedded in paraffin wax. The sections, which are 5–6 μm thick, were then prepared using rotary microtome (Leica RM 2125 RTS, Singapore) and stained with hematoxylin and eosin dye for microscopic observation of histopathological changes in the liver. Liver sections were scored and evaluated according to the severity of the hepatic injury as described by El-Beshbishy et al. [18].

### Phytochemical screening and HPLC profiling of MEMM

#### Phytochemical screening of the MEMM

The phytochemical screening of MEMM was performed according to the standard screening tests as adopted by Zakaria et al. [12]. The respective test, performed to detect alkaloids, flavonoids, triterpenes, tannins, saponins and steroids, was carried out based on 5.0 g of dried powder material and 100 mg of extract (organic) as given below.

##### Alkaloids test

Samples were soaked in chloroform followed by addition of ammoniacal chloroform. The mixture was then treated with sulphuric acid 10% and further tested with Mayer’s reagent. Formation of white precipitates indicates the presence of alkaloids.

##### Flavanoids test

The methanolic extract of samples were dissolved in ether and shaken in 10% ammonia solution. Formation of yellow colour in ammonia layer indicates the presence of flavonoids.

##### Triterpenes/steroids test

The methanolic extract of sample was analysed using Liebermann-Buchard reagent. The extract was mixed with few drops of acetic anhydride, boiled and cooled. Concentrated sulphuric acid was then added from the sides of the test tube and observed for the formation of a brown ring at the junction of two layers. Green coloration of the upper layer and the formation of deep red color in the lower layer would indicate a positive test for steroids and triterpenoids, respectively.

##### Tannins and polyphenolic compounds test

The methanolic extract of samples was mixed with 1% ferric solution. Formation of blue black colour indicates the presence of hydrolysable tannins, while brownish-green indicates that of condensed tannins.

##### Saponins test

The methanolic extract of samples was mixed with distilled water in a test tube. Formation of stable froth for at least 15 minutes indicates the presence of saponins.

#### HPLC profiling of the MEMM

The HPLC profiling of MEMM was carried out according to the methods described by Zakaria et al. [7] with slight modifications. Approximately 10 mg of MEMM was dissolved in 1 ml methanol. The solution was filtered through the membrane filter (pore size 0.45 μm) prior to analysis. HPLC profiling of the extract was carried out at the Laboratory of Phytomedicine, Forest Research Institute of Malaysia, Kepong, Malaysia. A Waters Delta 600 with 600 Controller and Waters 2996 Photodiode Array (Milford, MA, USA) equipped with an autosampler, online degasser and column heater was used for HPLC analysis. Data was analysed and processed using the installed Millenium 32 Software (Waters Product). The samples were separated at 27°C on a minibore Phenomenex Luna 5 μm C_18_ column with dimensions 250 x 4.60 mm using a one-step linear gradient. The solvents were (A) 0.1% aqueous formic acid and (B) acetonitrile and the elution system was as follows: Initial conditions were 85% A and 15% B with a linear gradient reaching 25% B at t = 12 min. This was maintained for 10 min after which the programme returned to the initial solvent composition at t = 25 min and continued for 10 min. The flow rate used was 1.0 ml/min and the injection volume was 10 μl. The HPLC was set at 254 and 336 nm.

### Identification of flavonoids in MEMM via HPLC analysis

In an attempt to identify the bioactive compound(s) present in MEMM, several standard flavonoids (i.e. quercetin, quercitrin and rutin) were injected into the HPLC system either alone or in combination with MEMM. The selection of flavonoid-based compounds was based on the range of wavelength of each peak obtained earlier.

### Statistical analysis

Data obtained are presented in mean ± standard error of mean (SEM). The data were analysed using the one-way analysis of variance (ANOVA) and the differences between the groups were determined using the Dunnet post hoc test with *P* < 0.05 as the limit of significance.

## Results

### Antioxidant activity of MEMM

The results of antioxidant potential of MEMM assessed using various antioxidant assays are shown in Table 1. The extract exerted a high antioxidant activity when assessed using the DPPH and superoxide scavenging assays with the percentage of antioxidant recorded in both test at values above 90%. In addition, the ORAC value recorded for MEMM was more than 27,000 μM Trolox Equivalent (TE)/100 g. Moreover, the MEMM also exhibited high TPC value, which is approximately two-fold of the minimum value (> 1000 mg gallic acid equivalent (GAE)/100 g) for a compound to be considered as having a high antioxidant value.

**Table 1:** The antioxidant profile and TPC value of MEMM, at the concentration of 200 μg/ml, as assessed using various oxidative assays

**Sample**	**DPPH radical scavenging (%)**	**Superoxide radical scavenging (%)**	**TPC (mg/100 g GAE)**	**ORAC (μM TE/100 g)**
**Sample concentration (μg/ml)**	200	200	200	**-**
**Standard**	**Ascorbic acid (AA) 200 μg/ml**	**Superoxide dismutase (SOD) 6X10**^**-3 **^**U/ml**	**Gallic acid (GAE) standard curve**	**Trolox standard curve**
**MEMM**	99.1 ± 0.5^h^	97.3 ± 0.3 ^h^	2610.7 ± 16.5	27, 662.0 ± 377.56

Note: For DPPH Radical Scavenging and Superoxide Scavenging: ^h^ high (70-100%). For TPC, the TPC value >1000 mg GAE/100 g is considered High Total Phenolic content. TPC- Expressed as milligram equivalent to Gallic Acid per 100 g of dry weight (mg GAE/100 g). For ORAC value, expressed as μM Trolox Equivalent (TE)/100 g, are mean values from triplicate wells in duplicate experiments, with SEM < 20%.

### In vivo hepatoprotective study

#### Effect of MEMM on the body weight and liver weights after induction with paracetamol

The administration of paracetamol following pretreatment with 10% DMSO (Group II) significantly (p < 0.05) increased the liver weight of rats when compared to the normal group (Group I) (Table 2). In contrast, pre-treatment with MEMM, at 250 (Group V) and 500 (Group VI) mg/kg, as well as 200 mg/kg silymarin (Group III), which is the reference hepatoprotective agent, significantly (p < 0.05) reduced the liver weight of paracetamol-treated rats. Moreover, significant (p < 0.05) reduction of the rats’ body weight was observed in groups pretreated with 250 (Group V) and 500 (Group VI) mg/kg of MEMM. Rats administered with 500 mg/kg MEMM (Group VI), as well as 200 mg/kg silymarin (Group III), showed significantly lower liver to body weight ratio when compared to Group II (Table 2).

**Table 2:** Effect of MEMM on the body and liver weights following treatment of rats against paracetamol

**Treatment**	**Dose (mg/kg)**	**Body weight (BW) (g)**	**Liver weight (LW) (g)**	**LW/BW (%)**
Control	-	211.0 ± 2.7	5.9 ± 0.3	2.9 ± 0.1
10% DMSO + Paracetamol	-	207.9 ± 3.6	8.7 ± 0.3^a^	4.9 ± 0.4^a^
Silymarin + Paracetamol	200	202.1 ± 2.6	6.9 ± 0.2^ab^	3.6 ± 0.1^ab^
MEMM + Paracetamol	50	203.5 ± 5.4	8.9 ± 0.2^a^	4.4 ± 0.1^a^
	250	193.6 ± 4.1^ab^	7.7 ± 0.1^ab^	4.6 ± 0.1^a^
	500	180.1 ± 3.9^ab^	6.2 ± 0.9^b^	3.8 ± 0.2^ab^

^a^ Data with this superscript differed significantly (P < 0.05) when compared to the normal control value in the respective column.

^b^ Data with this superscript differed significantly (P < 0.05) when compared to the 10% DMSO + Paracetamol-treated group in the respective column.

Values are expressed as means ± S.E.M. of six replicates.

#### Macroscopic and microscopic study of the paracetamol-induced toxic liver with and without pretreatment with MEMM

**Figure 1:**
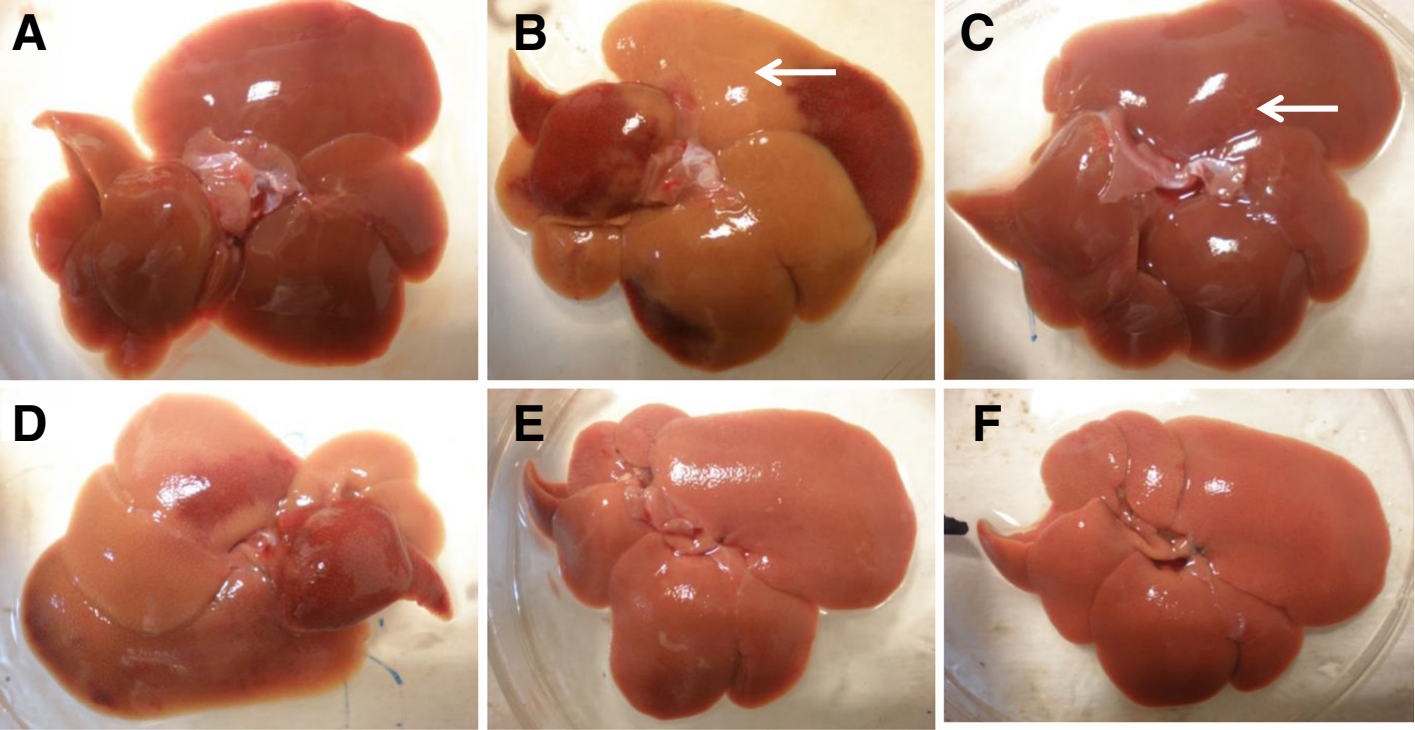
**Macroscopic observations of untreated and treated liver of rats. A)** normal liver, **B)** liver intoxicated with 3 g/kg paracetamol: gross image shows large area of discoloration (arrow), **C)** liver pre-treated with 200 mg/kg silymarin and induced with paracetamol: tiny hemorrhage spot was noted (arrow), **D)** liver pre-treated with 50 mg/kg MEMM and induced by paracetamol, **E)** liver pre-treated with 250 mg/kg MEMM and induced by paracetamol, **F)** liver pre-treated with 500 mg/kg MEMM and induced by paracetamol.

The liver collected was subjected to macroscopic and microscopic investigations to detect irregularities or abnormalities of the structure due to paracetamol. Macroscopic analysis of the liver intoxicated by paracetamol (Group II) demonstrated major brown color changes of the liver lobes (Figure 1B) in comparison to the liver of the normal group (Group II) with normal appearances (i.e. dark maroon in-color liver with a smooth surface) (Figure 1A). Pre-treatment with 200 mg/kg silymarin (Group III) reversed the toxic effect of paracetamol with spots of brown color changes observed (Figure 1C). Only pretreatment with MEMM, at the dosages of 250 (Group V) and 500 (Group VI) mg/kg, reversed the paracetamol -induced liver damage (Figure 1D-F).

**Figure 2:**
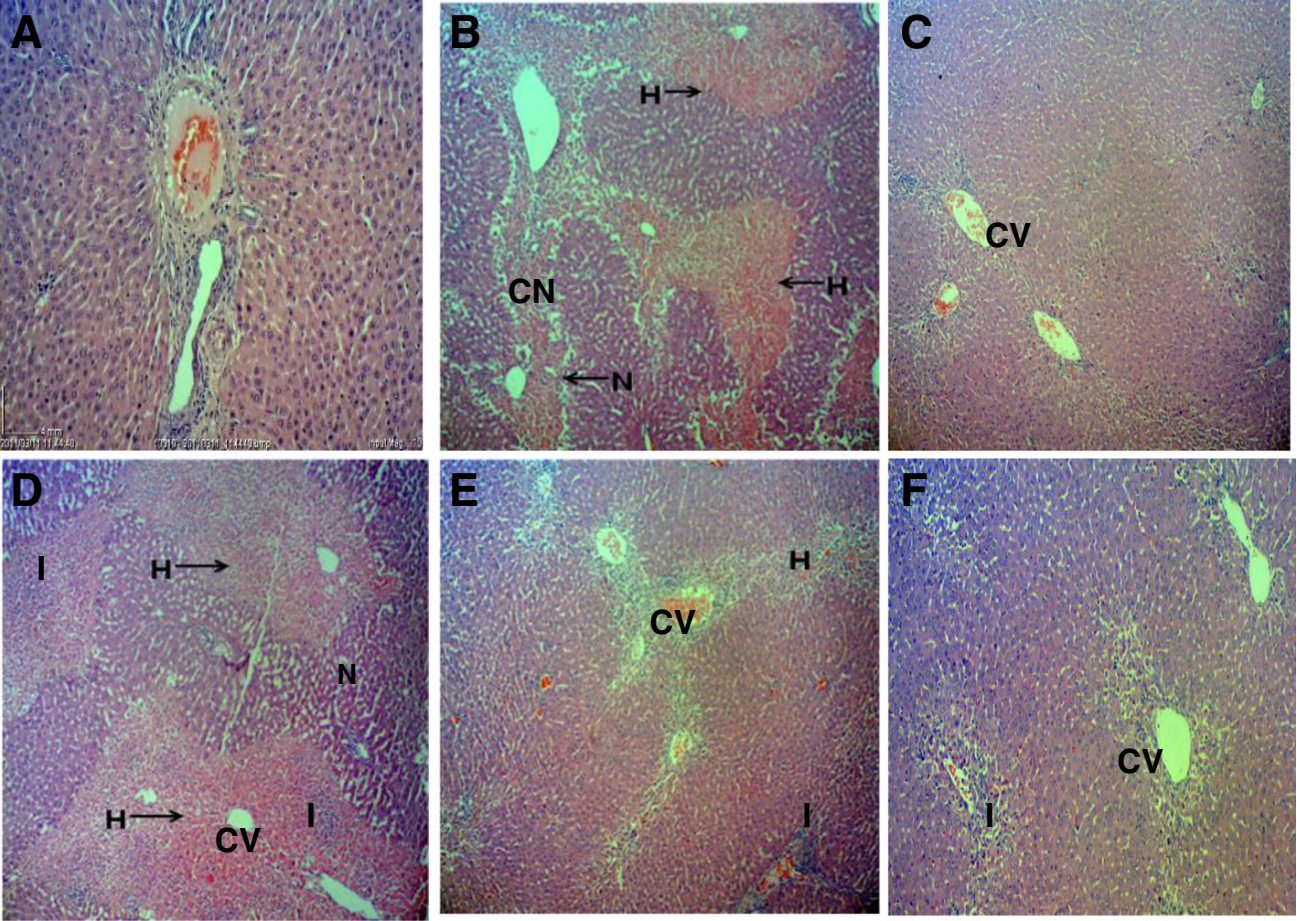
**Microscopic observations of untreated and treated liver of rats. A)** Normal, **B)** Section of liver tissue of 3 g/kg paracetamol-treated group (p.o) showing massive coagulative necrosis, haemorrhage and inflammation. **C)** Section of 200 mg/kg of silymarin liver tissue pretreated on the liver followed by paracetamol showing preservation of normal hepatocytes. **D)** Section of pre-treated 50 mg/kg MEMM liver tissue followed by paracetamol showing tissue necrosis, inflammation and haemorrhage. **E)** Section of pre-treated 250 mg/kg MEMM liver tissue followed by paracetamol showing mild haemorrhage and inflammation. **F)** Section of pre-treated 500 mg/kg MEMM followed by paracetamol showing normal histology with mild inflammation. (40x magnification). CV – central vein; CN – coagulative necrosis; I – inflammation; H – haemorrhage.

Microscopically, the non-paracetamol-intoxicated group (normal control; Group I) exhibited normal lobular architecture wherein the cytoplasm is well preserved with the normal hepatic cells of which a well defined sinusoids line and nucleus around the perivenular area are observed (Figure 2A). In comparison to Group I, the paracetamol-intoxicated liver after pre-treated with 10% DMSO (Group II) reveals massive coagulative necrosis, infiltration of lymphocytes, inflammation and haemorrhage at the perivenular, and midzonal region with periportal sparing (Figure 2B). Pretreatment with 200 mg/kg silymarin (Group III) preserved the normal architecture of hepatocytes and less signs of liver damages were seen (Figure 2C). The pathological changes due to the administration of paracetamol were found to reduced astonishingly as the dosages of MEMM increased. The 50 mg/kg MEMM (Group IV) failed to attenuate the paracetamol-induced liver toxicity (Figure 2D), whereas the 250 mg/kg MEMM (Group V) reversed the severe toxicity to mild hemorrhage and inflammation (Figure 2E) and the 500 mg/kg MEMM (Group VI) returned the toxic liver architecture to normal appearance with the presence of mild inflammation at the perivenular zone (Figure 2F). Overall, histopathological findings and their qualitative scoring are summarized in Table 3.

**Table 3:** Histopathological scoring of the paracetamol-induced liver injury pre-treated with various doses of MEMM in rats

**Treatment**	**Dose (mg/kg)**	**Steatosis**	**Necrosis**	**Inflammation**	**Haemorrhage**
Control	-	-	-	-	-
10% DMSO + Paracetamol		-	+++	++	++
Silymarin + Paracetamol	200	-	+	+	+
MEMM + Paracetamol	50	-	++	+	++
	250	-	+	+	+
	500	-	-	+	+

The severity of various features of hepatic injury was evaluated based on those following scoring scheme: - normal, + mild effect, ++ moderate effect, +++ severe effect.

#### Findings on liver serum enzymes level

Oral treatment with paracetamol following the 10% DMSO pretreatment (Group II) significantly (P < 0.05) elevated the ALT, AST and ALP serum marker level as compared to the normal group (non- paracetamol-intoxicated group; pre-treated with 10% DMSO; Group I) (Table 4). Interestingly, oral pre-treatment with 200 mg/kg silymarin (Group III) or, 250 (Group V) and 500 (Group VI) mg/kg MEMM demonstrated significant (P < 0.05) hepatoprotective activity against the paracetamol-induced liver damage as indicated by the decrease in level of those enzymes.

**Table 4:** Effect of paracetamol and protective treatments at ALT, AST and ALP (U/L)

**Treatment**	**Dose (mg/kg)**	**ALT (U/L)**	**AST (U/L)**	**ALP (U/L)**
Control	-	15.8 ± 2.9	95.1 ± 5.9	115.6 ± 7.0
10% DMSO + Paracetamol	-	1714.0 ± 142.2^a^	2266.0 ± 140.4^a^	345.8 ± 39.3^a^
Silymarin + Paracetamol	200	693.4 ± 162.5^ab^	651.2 ± 141.1^ab^	190.0 ± 9.6^ab^
MEMM + Paracetamol	50	1453.0 ± 125.9^a^	2200.7 ± 247.0^a^	384.0 ± 43.8^a^
	250	1308.0 ± 184.1^ab^	1719.0 ± 160.7^ab^	217.2 ± 17.2^ab^
	500	979.5 ± 174.0^ab^	1458.0 ± 271.2^ab^	178.2 ± 33.9^ab^

^a^ Data with this superscript differed significantly (P < 0.05) when compared to the normal control value in the respective column.

^b^ Data with this superscript differed significantly (P < 0.05) when compared to the 10% DMSO + Paracetamol-treated group in the respective column.

Values are expressed as means ± S.E.M. of six replicates.

### Phytochemical constituents of MEMM in comparison to its dried leaves and its HPLC profile

#### Pyhtochemical constituents of MEMM

The phytochemical constituents of the dried leaves and MEMM are shown in Table 5. Both the leaves and extract contained flavonoids, triterpenes, tannins, saponins and steroids, but not alkaloids.

**Table 5:** **Phytochemical screening of *M. malabathricum *** **leaves in powder form and MEMM**

**Class of compound**	**Sample**	
	**Dried leaf**	**MEMM**
Flavonoids	**++**	**+++**
Triterpenes	**++**	**++**
Tannins	**++**	**++**
Saponins	**++**	**+**
Steroids	**+++**	**++**
Alkaloids	**-**	**-**

For flavonoids, tannins, triterpenes and steroids – + : weak colour; ++ : mild colour; +++ : strong colour.

For saponins – + : 1–2 cm froth; ++ : 2–3 cm froth; +++ : >3 cm froth.

For akalioids – + : negligible amount of precipitate; ++ : weak precipitate; +++ : strong precipitate.

#### HPLC profile of MEMM

**Figure 3:**
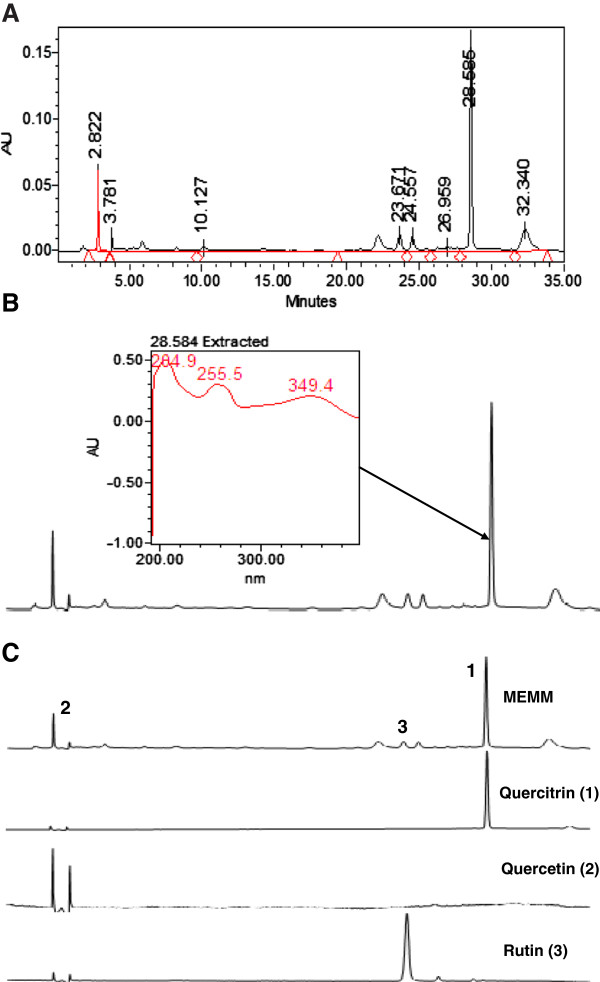
**The HPLC analysis of MEMM. A)** The HPLC profile of MEMM at the wavelength of 366 nm. **B)** The UV spectra analysis of MEMM at 366 nm. The chromatogram demonstrated the presence of several peaks, with the most major peak detected at the retention time (RT) of 28.584 min. This peak was observed at the λmax within the region of 284.9-349.4 nm, suggesting, in part, the presence of flavonoid-based compounds. **C)** The HPLC chromatogram of MEMM shows the presence of quercitrin (1) and rutin (2) at 366 nm.

The HPLC profile of MEMM at the wavelength of 254 and 366 nm is shown in Figure 3A. The best isolation of all detected peaks (4 major peaks) was observed at the wavelength of 366 nm. The four major peaks appeared in the chromatogram at the 366 nm wavelength tested at retention times of 2.75, 6.03, 7.44, 15.33 and 31.72 min. Further analysis demonstrated that the four peaks showed λ_max_ values in the region of 234.9, 254.3-367.2, 204.9-348.2 and 255.5-369.4 nm, respectively (Figure 3B).

### Identfication of flavonoids in MEMM via HPLC analysis

Based on the range of wavelength of each peak obtained following HPLC analysis of MEMM, several standard flavonoids, namely quercetin, quercitrin and rutin, were selected and used in the HPLC analysis in an effort to identify the bioactive compound(s) presence in the extract. Interestingly, the MEMM was found to contain quercetin, quercitrin and rutin (Figure 3C).

## Discussion

Paracetamol-induced liver injury model is a conventional model used to investigate the hepatoprotective activity of extracts/compounds [19]. In the present study, paracetamol (3 g/kg)) increased the liver weight and liver/body weight ratio of rats as previously reported and, upon biochemical analysis of the blood samples, increased ALT, ALP and AST, the liver enzymes in the serum. Histopathological observation of paracetamol-treated liver tissues revealed severe destruction of the liver normal architecture accompanied by reduction in the number of viable cells. Other observations include massive necrotic cells around the centrilobular zone expanding to parenchymal zone, which is distinguished by pyknosis and karyolysis nuclear.

Various mechanisms may be linked to the damage of the liver by different toxins. For examples, the CCl_4_ is transformed by the cytochrome P450 system to produce highly reactive trichloromethyl free radicals whereas upon the administration of high dose of paracetamol, the sulfation and glucuronodation routes become saturated causing the high percentage of paracetamol molecules to be oxidized to highly reactive N-acetyl-p-benzoquinone imine (NAPQI) [20]. NAPQI, in particular, binds covalently to hepatocellular macromolecules [21,22] that lead to activation of several biochemical processes (e.g. oxidative stress and glutathione (GSH) depletion) resulting in hepatotoxic effect [23]. NAPQI also acts on DNA, proteins, cellular proteins causing the dysfunction and death of hepatocytes that leads to liver necrosis [24]. Moreover, NAPQI bind to GSH to form conjugates, which will oxidize and convert GSH to glutathione disulfide (GSSG). This, in turn, reduces the GSH level in blood and liver [25]. The depletion of GSH level in blood and liver triggers the mitochondrial dysfunction, increase of lipid peroxidation and, lastly, development of acute hepatic necrosis. The necrotized hepatic parechymal usually releases pool of several enzymes, such as AST and ALT into the circulation [26] that is regarded as specific enzyme for detection of liver abnormalities [27,28]. Moreover, AST is also considered as an essential marker as it is susceptible to mitochondrial deformation primarily in zone 3, centrilobular zone [27,28].

The present study demonstrated the ability of MEMM to exert hepatoprotective activity and supported our earlier hypothesis that the extract possessed anti-inflammatory, antioxidant activities and also hepatoprotective effects. The hepatoprotective potential of MEMM was further supported by the ability of the extract to reduce the serum liver enzymes (ALT and AST) level and the liver/body weight ratio, and the histopathological findings. However, through this study also the consumption of MEMM for 7 days was found to reduce the body weight of rats. This finding is in accordance with previous studies by Shimoda et al. [29] and Auvichayapat et al. [30], who reported on the presence of extracts such as green tea extract or green bean coffee extract, that reduced body weight of rats, respectively. Although the exact compound(s) that caused the suppression of rats’ body weight was not yet determined and not part of the objective of the present study, the presence of high polyphenolic compounds as well as flavonoids in MEMM [12] is suggested to play a significant role based on previous report by Yang et al. [31] and Pichiah et al. [32]. According to Shimoda et al. [29] the body weight loss effect is usually seen with extracts containing high polyphenols (i.e. green tea extract and coffee). Previous study demonstrated that that modest weight loss, which is approximately 5-10% of the initial body weight, is correlated with marked advancements in various risk factors [33]. The ability of MEMM to trigger a body weight loss effect as seen in the present study is suggested to cause no adverse effect to the body because MEMM considerably prevented the increase in body weight of approximately 10%. Since MEMM is a crude extract containing several types of compounds, including quercetin, quercitrin and rutin, it is also plausible to propose that the ability to reduce rats’ body weight involves synergistic action of those compounds as reported by Yang et al. [31].

Lately, much interest has been given towards the possible involvement of oxidative stress in the initiation and/or progression of paracetamol-induced hepatotoxicity. Reactive oxygen and nitrogen intermediates, generated by hepatic paranchymal and non-paranchymal cells are thought to play important roles that lead to paracetamol-induced injury [34-36]. Therefore, any compounds with high antioxidant capability are potential candidate for further development as hepatoprotective agents. Of various potential candidates, plants have been prominent source of either new or known extracts and bioactive compounds with diverse pharmacological activities. Plants have been reported to exert antioxidant activity whereas antioxidant activity has been suggested as the possible mechanism responsible for the protection against paracetamol-induced liver injury. Interestingly, our hypothesis is in accordance with report made by Gupta et al. [37] that the combination of hepatoprotective and antioxidant activities synergistically exerts the processes of initiation and progress of hepatocellular damage. In the present study, the MEMM has been shown to possess a high antioxidant effect through the DPPH- and superoxide anion- radical scavenging, and ORAC assays. Moreover, the extract also contained a very high TPC value wherein several reports have demonstrated the correlation between the high TPC value and the high antioxidant potential [38,39]. Therefore, it is plausible to suggest that the mechanism of hepatoprotection exhibited by MEMM may, in part, include its ability to self-act as a free radical scavenger that might intercept those free radicals involved in paracetamol metabolism by the microsomal enzymes [23]. Therefore, by ensnaring oxygen related radicals the MEMM could hamper their interaction with polyester fatty acids and would eliminate the enhancement of lipids peroxidative processes leading to MDA formation [36-39]. With regards to the contribution of the bioactive compounds towards the observed antioxidant activity of MEMM, the involvement of quercetin, quercitrin and rutin are worth mentioning based on previous reports [40-42].

Other than the oxidation processes, the inflammatory processes have been thought to be intimately involved in the chemical-induced hepatotoxic processes [43]. The inflammatory processes activated by toxic agents like PCM produce various mediators, which are involved in the production of ROS and NO that can affect liver damage or repair. Therefore, it is likely to propose that the extracts/compounds exerting an anti-inflammatory activity may also show hepatoprotective activity. Based on the histological observations and scoring of the hepatotoxic liver, pre-treatment with MEMM reduced the appearance of inflammation suggesting the involvement of anti-inflammatory mechanism [6,44]. The potential of the bioactive compounds in the anti-inflammatory activity of MEMM could be contributed by quercetin, quercitrin and rutin, in accordance with previous reports [45-47].

Phytochemical constituents have been known to play important role in determining the pharmacological potentials of various medicinal plants. Various types of bioactive compounds have been isolated, identified and scientifically proven to possess certain types of pharmacological activities from various types of plants. In term of the MEMM, flavonoids, tannins, saponins and steroids have been identified in the extract, which has also been reported to possess high TPC value [12]. The presence of flavonoids and tannins [48], triterpenoids and flavonoids [49,50], flavonol [51], flavonoids [52], triterpene, amides and flavonoids [53], flavonoids, phenolics, triterpenes, tritepenoids, tannins, saponins and steroids [54] from the leaves of *M. malabathricum* extracted using various organic solvents extracts have been reviewed elsewhere [8]. In term of the pharmacological activities, flavonoids exhibited antioxidant [55,56], anti-inflammatory [57] and hepatoprotective [56,57] activities; condensed tannins possess free radical scavenging and antioxidant, anti-inflammatory and hepatoprotective activities [58]; saponins exhibited hepatoprotective activity via modulation of its antioxidant [59] and anti-inflammatory activities [60]. Based on all of the above reports, the MEMM-induced hepatoprotective activity is believed to involve synergistic action of, at least, flavonoids, saponins and condensed tannins. The HPLC analysis of MEMM at 366 nm demonstrated the presence of 4 major peaks with λ_max_ falling in the region of 234.9, 254.3-367.2, 204.9-348.2 and 255.5-369.4 nm. These peaks may represent flavonoid-types of bioactive compounds (e.g. flavonoids subgroups are flavones, flavanones, flavonols, dihydroflavonols, and flavanonols) (Ali et al., 2010). The UV–vis spectra of flavonoids consist of two absorbance bands labeled as band A and band B [61]. For flavones and flavonols, band A falls in the range of 310–350 nm and 350–385 nm while band B lies in the range of 250–290 nm, respectively. In the case of flavanones and dihydroflavonols, the wavelength of band A is often in the range of 300–330 nm while band B falls in the range of 277–295 nm. Furthermore, many polyphenols including flavonols demonstrated maximal absorbance at the wavelengths between 270 and 290 nm [52]. Based on their respective wavelength in the chromatogram, we suggested that peak 2 and peak 4 might probably be the flavonoid-types of compounds. Further HPLC analysis in preliminary attempt to standardize the extract demonstrated that the MEMM contained quercetin, quercitrin and rutin.

## Conclusion

The MEMM exhibited hepatoprotection against paracetamol-induced liver injury model, which could be, partly, attributed to its antioxidant activity and, linked to the presence and synergistic action of flavonoids, tannins and saponins. Further studies are warranted and are being planned to determine the possible hepatoprotective mechanism(s) involved and, to isolate and identified the responsible bioactive compounds.

## Competing interests

The authors declare that they have no competing interest.

## Authors’ contribution

SSM, MFFK, and FY carried out the animal studies, biochemical analysis and drafted the manuscript. NDM, MSS and KFJ carried out the antioxidant studies, phytochemical screening, HPLC analysis and helped in the preparation of manuscript. NM involved in the macroscopic and microscopic analysis and helped to draft the manuscript. SMC involved in the statistical analysis and and helped to draft the manuscript. DS and MT helped in the phytochemical and HPLC works. ZAZ conceived of the study, participated in its design and helped to draft the manuscript. All authors read and approved the final manuscript.

## Pre-publication history

The pre-publication history for this paper can be accessed here:

http://www.biomedcentral.com/1472-6882/13/326/prepub
